# Closer to a Uniform Language in Colposcopy: Study on the Potential Application of 2011 International Federation for Cervical Pathology and Colposcopy Terminology in Clinical Practice

**DOI:** 10.1155/2017/8984516

**Published:** 2017-05-25

**Authors:** Yanyun Li, Xiaoling Duan, Long Sui, Fengying Xu, Shuifang Xu, Hongwei Zhang, Congjian Xu

**Affiliations:** ^1^Medical Center of Diagnosis and Treatment for Cervical Diseases, Obstetrics and Gynecology Hospital, Fudan University, Shanghai 200011, China; ^2^Department of Obstetrics and Gynecology, Jinshan Hospital, Fudan University, Shanghai 201508, China; ^3^Department of Obstetrics and Gynecology, Tinglin Hospital, Shanghai 201505, China; ^4^Department of Gynecology, Obstetrics and Gynecology Hospital, Fudan University, Shanghai 200011, China; ^5^Department of Obstetrics and Gynecology of Shanghai Medical School, Fudan University, Shanghai 200032, China; ^6^Shanghai Key Laboratory of Female Reproductive Endocrine Related Diseases, Shanghai 200011, China; ^7^Institute of Biomedical Sciences, Fudan University, Shanghai 200032, China

## Abstract

As the newest colposcopic terminology, the 2011 International Federation for Cervical Pathology and Colposcopy (IFCPC) classification provides standardized interpretation of colposcopic findings. In this study, we analyzed the colposcopic accuracy and the significance of individual findings according to the 2011 IFCPC classification in 525 patients, reviewed by 13 trained colposcopists. Results show that colposcopic diagnoses are in 64.95% perfect agreement with cervical pathology, with 63.64% sensitivity and 96.01% specificity for high-grade squamous intraepithelial lesion (HSIL+). And the accuracy is reproducible across different experienced examiners. Many individual findings, especially the two new signs, inner border sign and ridge sign, are proved to have good predictive accuracy, while iodine negativity demonstrates an inferior performance. However, the distribution of three cervical transformation zone (TZ) types is heterogeneous in examiners. A comparison was also made of the findings of another two colposcopists without nomenclature training according to the Reid Colposcopic Index (RCI), modified RCI, and Swede Score. Results show that colposcopic accuracies in them are lower than in those nomenclature trained colposcopists. The 2011 IFCPC nomenclature improves colposcopic accuracy in trained colposcopists, like speaking the same language. However, the reproducibility of TZ and the predictive value of a few signs remain to be discussed.

## 1. Introduction

In recent years, with HPV detection being widely used in cervical screening, colposcopy has become increasingly common. Accurate colposcopy can substantially reduce the number of blinded 4-quadrant cervical biopsies and unnecessary conizations and contribute to invasive surgical procedures. However, colposcopy is considered a subjective procedure that is highly dependent on the observer's evaluation; therefore, how to standardize its evaluation has always been the subject of concern and discussion. A variety of colposcopic scoring systems have been introduced and related to lesion colors, margins, vascularization, and appearance after the application of acetic acid and Lugol iodine solution. Examples commonly used include the Reid Colposcopic Index (RCI) [1], the modified Reid Colposcopic Index [2], and the Swede Score [3]. In all of them, cutoff scores were set for different cervical lesion grades, which greatly simplify the evaluations and make them easy to learn. However, the correlation between them and histopathology has been shown to be unsatisfactory in many studies [4–9].

To improve the accuracy of colposcopy, repeated efforts have been made to unify the colposcopic nomenclature which provides standardized interpretations of the colposcopic findings and organizes comprehensive classification. In 2011, the International Federation for Cervical Pathology and Colposcopy (IFCPC), based on the versions developed in 1975 [10], 1990 [11], and 2002 [12], presented the new international colposcopic terminology/classification system at the 14th World Congress in Rio de Janeiro and recommended that the 2011 terminology should replace all previous colposcopic nomenclature [13]. Nomenclature emphasizes the significance of various normal or abnormal colposcopic findings more than colposcopic indexes did. Examples include the patterns of cervical transformation zone (TZ), as well as the distinction between lesions located inside and outside the TZ. Compared to previous versions, the 2011 nomenclature was further improved to provide a general assessment of the examination, detailed and refined the characteristics of findings with some newly introduced signs added, and introduced for the first time the types of excision [13], which represent the latest understanding globally on precancerous lesions of the lower genital tract in women.

Although this IFCPC document presents descriptive terminology rather than a colposcopic index, the classification clearly indicates which patterns of colposcopic findings should be suspected as a benign cervix, low-grade lesions, high-grade lesions, and invasive cancer. The accuracy of the preceding 2002 nomenclature diagnosis and its reproducibility have been analyzed in some reports and good results were obtained [14, 15]. However, as for the applicability of the 2011 IFCPC terminology, evaluation studies are extremely scant [16, 17].

In this study, we analyzed the colposcopic accuracy according to the 2011 IFCPC classification in nomenclature trained colposcopists by comparison with cervical histopathology and assessed the significance of the individual colposcopic findings in nomenclature. And we further made a comparison with the colposcopists without nomenclature training according to three colposcopic indexes.

## 2. Materials and Methods

### 2.1. Subjects and Procedure

We performed a retrospective analysis on the colposcopic diagnoses of patients from September 2014 to February 2015 in the colposcopy clinic at the Obstetrics and Gynecology Hospital of Fudan University, Shanghai. A total of 525 women referred to the colposcopy clinic with suspicious-looking cervixes, abnormal cervical cytology, or positive high-risk HPV testing (Hybrid Capture II or Cobas HPV test) were included in the study. Suspicious-looking cervixes include abnormal bleeding cervixes (or obvious contact bleeding), abnormal vaginal discharge cervixes, recurrent erosion cervixes, cervical polyp, leukoplakia, condyloma, gross neoplasm, irregular surface, cervical canal stenosis, or barrel-like cervixes. Cytologic abnormalities include atypical squamous cells of undetermined significance (ASC-US) or worse. The women who had obtained results of cervix pathology within 1 year or had a history of hysterectomy or pelvic radiotherapy or any ablative treatment or excision of cervical lesions (such as LLETZ, laser) or those who had an inadequate colposcopy or with incomplete data and those who only underwent colposcopic observation but had no histopathologic diagnosis were excluded from the analysis.

They received colposcopic diagnoses according to the 2011 IFCPC classification by one of the 13 colposcopists who have received colposcopic training for 2011 IFCPC nomenclature system and were certified by the CSCCP (Chinese Society for Colposcopy and Cervical Pathology). Directed punch biopsies were performed on the abnormal areas. In cases where the colposcopy did not reveal any lesions but was unsatisfactory, four-quadrant biopsies from the squamocolumnar junction (SCJ) endocervical curettage may be taken. If the colposcopy was satisfactory and did not reveal any lesions, the patient may not undergo biopsy and may be excluded from the analysis. Furthermore, digital colpophotographs of each patient were reviewed by another two colposcopists who were also certified by the CSCCP but did not receive nomenclature training, who were blinded to the patients' previous colposcopic diagnoses, according to the RCI, modified RCI, and Swede Score. Neither the colposcopists nor the pathologists were blinded to the referral cytologic and HPV test results.

Leisegang BG/LED Y/C optoelectronic integrated digital colposcopy (Leisegang Feinmechanik Optik GmbH, Berlin, Germany) was utilized, and images were obtained by a Canon EOS600D camera. The cervix was examined in 3 steps according to the following standard protocol: (1) without the use of reagents, (2) 1 minute after the application of 5% acetic acid, and (3) immediately after the application of Lugol iodine solution.

The sample size was calculated according to a sensitivity of 60% of the colposcopic diagnosis, which was determined during the pilot study [18]. We accepted Type 1 error at 5%; the minimum sample size was calculated to be 369 patients.

All women provided written informed consent to participate in the study, which was approved by the ethical committee of the hospital.

### 2.2. IFCPC Classification Colposcopic Diagnosis

According to the 2011 nomenclature, detailed colposcopic impressions with individual findings were documented as follows [13]. (1) The first is general assessment: adequate or inadequate for the reason, SCJ visibility, and three types of TZs. Type 1 is entirely located on the ectocervix. Type 2 involves the endocervical canal, but the SCJ is still visible. Type 3 shows endocervical involvement without a fully visible SCJ. (2) Second is colposcopic description: original squamous epithelium, columnar epithelium, metaplastic change, deciduosis, and location and size of lesions. (3) Third is graded findings: thin acetowhite epithelium (AWE), fine mosaic and fine punctuation as minor changes, suggestive of low-grade disease; dense AWE, coarse mosaic, coarse punctuation, sharp border, inner border sign, and ridge sign as major changes, suggestive of high-grade disease; leukoplakia, erosion, and iodine negativity as nonspecific changes; atypical vessels and additional signs (exophytic lesion, necrosis, ulceration, etc.) suspicious for invasion changes; condyloma, polyps, and obvious contact bleeding as miscellaneous findings. (4) Finally, colposcopists proposed a hypothetical diagnosis based on the criteria above, which were classified as normal or benign, low-grade squamous intraepithelial lesion (LSIL), high-grade squamous intraepithelial lesion (HSIL), and invasive carcinoma [13]. It should be noted that the same colposcopic finding could be presented in different lesion grades. In doubtful diagnosis, it was reviewed by another certified senior colposcopist, who decided on the final colposcopic diagnosis.

### 2.3. Colposcopic Diagnosis Using Other Scoring Systems

The RCI has a total score of 0–8 with 4 items added based on the following 4 colposcopic features: color, lesion margin, vessel pattern, and iodine staining. The cutoff values for LSIL and HSIL were ≥3 and ≥5, respectively [1, 19–21]. The modified RCI has a total score of 0–6, which omits iodine staining. The cutoff values for LSIL and HSIL were ≥1 and ≥3, respectively [2, 22]. The Swede Score adds the lesion size as a 5th variable and has a total score of 0–10. The cutoff values for LSIL and HSIL were ≥5 and ≥8, respectively [3, 23].

### 2.4. Pathological Diagnosis

According to the 2012 Lower Anogenital Squamous Terminology (LAST), the histopathological diagnoses were classified as normal or benign, LSIL, HSIL, and carcinoma (including microinvasive carcinoma and invasive carcinoma), which were taken as the “gold standard” [24].

### 2.5. Statistical Method

Data analysis was performed by using the Statistical Package for the Social Sciences Version 19.0 Software (SPPS 19.0). The estimative agreement between colposcopic diagnosis and cervical histopathology was determined using weighted *κ* statistics, perfect agreement, agreement within one grade, overestimated and underestimated. The criteria used to judge the strength of agreement in *κ* value were 0.0–0.20 (slight), 0.21–0.40 (fair), 0.41–0.60 (moderate), 0.61–0.80 (substantial), and 0.81–1 (almost perfect). The sensitivity, specificity, positive predictive value (PPV), negative predictive value (NPV), positive likelihood ratio (PLR), negative likelihood ratio (NLR), and Youden Index (YI, sensitivity + specificity − 1) were used to assess the accuracy.

Correlations between categorical variables were calculated by using *χ*^2^ test or Fisher's exact test, whereas difference comparisons between paired categorical variables were calculated by using McNemar test. Difference between two categorical variables adopts multiple comparisons. Confidence intervals (95% CI) were calculated where appropriate. Any *P* value less than 0.05 was considered statistically significant.

## 3. Results

### 3.1. Agreement between Colposcopy and Histopathology

A total of 525 patients (40.13 ± 10.23 years) were included in this study. In the nomenclature trained colposcopists, the perfect agreement between the IFCPC classification colposcopic diagnosis and the histopathology was 64.95%, with consistency of kappa = 0.436 (*P* < 0.001, 95% CI = 0.370–0.502), and the agreement within one grade was 97.14% (Table 1). In the colposcopists without nomenclature training, the agreements between the RCI, the modified RCI, and the Swede Score were 57.14%, 32.95%, and 56%, respectively; the kappa values were 0.272 (*P* < 0.001,* 95% CI = 0.201*–*0.336*), 0.096 (*P* < 0.001,* 95% CI = 0.057*–*0.135*), and 0.146 (*P* < 0.001,* 95% CI = 0.091*–*0.208*); agreements within one grade were 94.1%, 94.67%, and 89.9%. With the IFCPC classification, overestimated diagnoses (20.38%) were approximately equal to underestimated diagnoses (14.67%), while in the other three approaches, the overestimated and underestimated diagnoses were 17.33% and 25.52%, 57.71% and 9.33%, and 4.95% and 39.05%.

### 3.2. Significance of the Individual Colposcopic Findings in the IFCPC Nomenclature

Based on the colposcopic records of nomenclature trained colposcopists, the distributions of the individual colposcopic findings of the 2011 terminology in 525 patients and their PPVs are analyzed (Table 2). Among all 854 abnormal findings (excluding normal colposcopy), 40.3% were diagnosed as normal/benign, 29.4% were diagnosed as LSIL, 28.1% were diagnosed as HSIL, and 2.2% were diagnosed as cancerous. For minor changes, the majority of fine mosaic (48%) and fine punctuation (35.5%) changes were related to LSIL, while 46% thin AWE existed in normal/benign cervixes. For major changes, the majority of dense AWE (68.3%), coarse mosaic (69.2%), coarse punctuation (58.8%), sharp border (38.5%), inner border sign (100%), and ridge sign (71.4%) were related to HSIL. A total of 48.5% of iodine negativity, 64.5% of polyps, and 62.5% of obvious contact bleeding existed in normal/benign cervix. All of the major findings had high PPV for HSIL+ (HSIL/carcinoma) lesions: dense AWE (73.0%), coarse mosaic (76.9%), coarse punctuation (64.7%), sharp border (53.8%), inner border sign (100%), and ridge sign (100%). Minor changes had low PPV for HSIL+ lesions but high PPV for LSIL+ (LSIL/HSIL/carcinoma) lesions, including thin AWE (54.0%), fine mosaic (88.0%), and fine punctuation (67.7%). Atypical vessels and additional changes had the highest PPV for carcinoma (100%). However, iodine negativity demonstrated an inferior performance (21.2% for LSIL+ and 51.5% for HSIL+). In the miscellaneous findings, condyloma indicated LSIL lesions (100%), while the others had no clear indicative meanings for lesions.

### 3.3. Comparison of Nomenclature Examiners with Different Experience

According to the level of examiner experience, we subdivided 13 IFCPC nomenclature trained colposcopists into three groups: more than 10 years (4 colposcopists), 5–10 years (6 colposcopists), and less than 5 years (3 colposcopists). Six initial colposcopies with doubtful diagnosis were reviewed by the senior colposcopist and finally counted into the more than 10 years group. And we compared the agreements between the colposcopic diagnosis according to 2011 IFCPC classification and histopathology among the three groups.  Table 3 shows that the agreements between the colposcopy and histopathology in the three groups were matched at 65.0%, 61.4%, and 70.0%. The differences between the groups were not statistically significant (*χ*^2^ = 2.696,* P* = 0.260).

### 3.4. Utility and Reproducibility of TZs in the IFCPC Nomenclature

We then analyzed the distribution of the three types of cervical TZ described in the IFCPC nomenclature among three age groups in 525 patients: ≤30 (98 patients), 30–50 (333 patients), and >50 years (94 patients). Types 1, 2, and 3 of TZs accounted for 22.29%, 7.24%, and 70.48% of all patients, respectively; 47.96%, 11.22%, and 40.81% of patients ≤30 years, respectively; and 21.02%, 7.80%, and 71.17% of patients 30–50 years, respectively. Type 3 accounted for most (98.94%) of the patients >50 years. The differences of the proportion in the three groups were statistically significant (*χ*^2^ = 80.48, *P* < 0.001). In order to test the reproducibility of TZs discrimination, we further calculated the frequency of the three TZs in different examiners (13 colposcopists), as shown in Type 1: 6.25%~35%; Type 2: 0.0%~20%; and Type 3: 55%~91.53% (Table 4). According to Chi-square test, the difference in the frequency was statistically significant (*χ*^2^ = 47.71, *P* < 0.01). Further multiple comparisons confirmed that the differences between Type 1 and Type 2 (*χ*^2^ = 23.358, *P* < 0.05), Type 1 and Type 3 (*χ*^2^ = 25.184, *P* < 0.05), and Type 2 and Type 3 (*χ*^2^ = 21.138, *P* < 0.05) were statistically significant.

### 3.5. Evaluating the Accuracy of the Colposcopic Diagnosis

The values of the colposcopic diagnoses as predictors of the histological diagnoses for HSIL+ and LSIL+ lesions are summarized in Table 5. In the nomenclature trained colposcopists, when taking HSIL as the cutoff, the sensitivity, specificity, YI, PPV, and NPV were 63.64%, 96.01%, 0.596, 78.75%, and 91.91%, respectively. However, in the colposcopists without nomenclature training, when the RCI was used, they were 38.38%, 95.54%, 0.339, 66.67%, and 86.97.%; when the modified RCI was used, they were 54.55%, 87.09%, 0.416, 49.54%, and 89.18%; when the Swede Score was used, they were 13.13%, 98.59%, 0.117, 68.42%, and 83%. When taking LSIL as the cutoff, the sensitivities in each approach were improved, the specificities were decreased, and most of the comprehensive indexes (YI, PLR, and NLR) were not as good as those when HSIL was used as the cutoff.

### 3.6. Comparison between the IFCPC Classification and the Three Scoring Systems

The comparison of the colposcopic diagnosis for HSIL+ between nomenclature trained colposcopists and those without nomenclature training is shown in Table 6, including the comparison between the IFCPC classification and the RCI, modified RCI, and Swede Score. According to McNemar's test, the difference between the IFCPC classification and the RCI score was statistically significant (*χ*^2^ = 134.65, *P* < 0.001). Likewise, significant differences also existed between the IFCPC classification and the modified RCI (*χ*^2^ = 184.69, *P* < 0.001) and the Swede Score (*χ*^2^ = 96.46, *P* < 0.001).

## 4. Discussions

Despite the various colposcopic scoring and grading systems, no consensus has yet been reached to standardize the colposcopic assessment [1–4]. As the newest international colposcopic terminology, the 2011 IFCPC nomenclature has been proposed for several years; however, the evaluation studies are extremely scant. In this study, we analyzed the clinical applicability of the new nomenclature in predicting cervical diseases. Although the agreement between histopathology and colposcopy according to the IFCPC classification in nomenclature trained colposcopists was shown to be only moderate, it is much better than those without nomenclature in many former studies [5–9], and the colposcopic accuracy was shown to be reproducible across different experienced examiners. Many individual findings in nomenclature were proved to have good predictive accuracy, while TZ types were of significant heterogeneity in examiners. Like speaking the same language, the nomenclature colposcopy in our study obtained a better sensitivity, specificity, PPV, and NPV for HSIL+, compared with three colposcopic indexes (RCI, modified RCI, and Swede Score) in colposcopists without nomenclature training.

Our results indicate that the perfect agreement, agreement within one grade, and strength of consistency between colposcopic diagnosis and cervical histopathology in nomenclature trained colposcopists according to the 2011 classification were all better than those in colposcopists without nomenclature training according to the RCI, modified RCI, and Swede Score. As the most well-known scoring system, the RCI was first reported by Reid and Scalzi in 1985 [1] and was shown to have good consistency of strength kappa as 0.55–0.74 with histopathology in several reports [19–21, 25, 26]. However, with cervical screenings and typical colposcopic impressions of precancers becoming increasingly uncommon, the accuracy of colposcopy as the standard method has been questioned [4]. An unsatisfactory correlation to histopathology has been shown in many studies, with an only 32%–37% perfect agreement, 75%–77% agreement within one grade, and only poor strength in the studies by Brotzman (*κ* = 0.26) [5], Baum (*κ* = 0.2) [6], and Massad (*κ* = 0.2) [7], which were approximately equal to that shown in our study using RCI in colposcopists without nomenclature training. Some scholars have used the modified RCI with iodine staining removed in colposcopic assessment. However, the results of the ASCUS LSIL Triage Study (ALTS) showed poor (*κ* = 0.17) agreement between the modified RCI and histopathology and poor reproducibility in colposcopists [2, 8, 9]. Likewise, the kappa value in our study showed only 0.096. The Swede Score was proposed by Strander in 2005, which highlighted the importance of lesion size and has a reported specificity of ≥8 scores for HSIL higher than 90% [3, 23]. Although the strength of the agreement of the 2011 classification in our study was only moderate, it was much better compared with that of the RCI, modified RCI, and Swede Score. Furthermore, the IFCPC classification was balanced in overestimated and underestimated diagnoses, in which overestimated diagnoses may increase the number of unnecessary cervical biopsies or conizations in clinical practice, while underestimated diagnoses lead to inadequate biopsy or inaccurate positioning. Instead, the other three methods were substantially unequivalent between them. This is especially the case with the modified RCI and Swede Score, where the underestimated diagnoses were rather high, consistent with approximately 1/3 of HSIL underdiagnosed colposcopies in other reports [27, 28].

In our study, the colposcopic sensitivity, specificity, PPV, and NPV for HSIL+ according to the IFCPC classification were all better than those using the RCI, modified RCI, and Swede Score. The advantage of the comprehensive indexes (YI, PLR, and NLR) was more obvious. Further calculations of significant differences confirmed the superiority. In various reports over the past 30 years, the sensitivity, specificity, PPV, and NPV of the RCI were shown to be extremely variable [1, 19–21, 25, 26], while the results of the modified RCI and Swede Score were roughly equal to ours [2, 3, 8, 9, 22, 23]. A few studies have reported the colposcopic accuracy of the IFCPC classification. The colposcopic sensitivity, specificity, PPV, and NPV for HSIL were reported to be 61.1%, 94.4%, 43.1%, and 77.9% in Hammes et al.'s study in 2007 when the 2002 nomenclature was used [14] and 84.8%, 66.1%, 10.3%, and 98.9% in Ghosh et al.'s study in 2014 when the 2011 nomenclature was used [16]. Our results with the 2011 nomenclature were appreciably better than the former study and showed lower sensitivity, higher specificity, much higher PPV, and equal NPV compared with the latter. In addition, the perfect agreement and consistency kappa with histology in our study were much better than in Ghosh's study. The possible reason for this lies in the different populations selected because Ghosh's study was conducted in a specific screened population of a community-based program. With LSIL as the cutoff, the comprehensive indexes in any method are not as good as those with HSIL as the cutoff, indicating that colposcopy is most accurate in identifying high-grade diseases no matter what assessment system is used [4, 20].

A characteristic of the 2011 nomenclature is various refined colposcopic findings. In this study, they were analyzed individually. Among all the 854 abnormal findings, 59.7% were related to abnormal histology (LSIL+), while this proportion of the 2002 nomenclature issued by Hammes et al. was only 39.7% [14]. In our study, most minor changes had a good PPV for LSIL, and all the major changes showed the highest PPV for HSIL. This was especially true for two newly introduced signs, inner border sign and ridge sign, as their PPV for HSIL reached 100%, although they were rather uncommon. Meanwhile, in the reports of the 2002 nomenclature, minor and major changes showed much lower PPV for LSIL and HSIL [14]. We conclude that colposcopic findings of new nomenclature were well matched with grading and better predictive accuracy for histopathology results than in previous versions. With the extensive spread of the IFCPC nomenclature and more detailed improvements in the new version, colposcopists received increasing recognition and confidence in normal/abnormal findings. It is noted that thin AWE were quite common but a majority of them were related to benign cervixes, and their PPV for LSIL is relatively lower than other minor changes. The same was true in many other studies: most women with AWE do not have severe lesions [9] and lesions with non-HPV16 oncogenic types do not appear as distinctly acetowhite [29]. Another common finding is iodine negativity; however, its predictive values were much lower than for minor and major changes. Unlike with the RCI, Swede Score, and previous IFCPC classifications, which take iodine negativity as an important variable or abnormal finding [1, 3, 10–12], the 2011 classification has reclassified it as a nonspecific finding. In the present study, this change was confirmed to be reasonable. In miscellaneous findings, some polyps (35.5%) were diagnosed as LSIL+, indicating a need for special attention in clinics, and most contact bleeding (62.5%) indicated inflammation.

One of the highlights of the IFCPC nomenclature is term cervical TZ between the original squamous epithelium and columnar epithelium within which varying degrees of maturity may be identified. The 2011 nomenclature proposed a general assessment for colposcopy examination by three variables, including the three TZ types, with the popular terms “satisfactory” and “unsatisfactory” replaced [13], and introduced for the first time the types of excision outlined in the Addendum. Similar to a multicenter study of the German colposcopy network including 3,761 patients [30], our results show that TZs were under different distribution due to age; the greater the age, the higher the proportion of Type 3, which was the most common type in women >50 years of age (98.94%). However, the distributions of Type 1 (22.29%) and Type 2 (7.24%) were much different from those in the German study (24% and 57%). We then found the frequency of the three TZs, and any two TZs showed great difference among the 13 colposcopists. Likewise, the German study showed significant heterogeneity of TZs in different clinics. Therefore, although the IFCPC has put forward the three types of TZ since the 2002 version on which evaluation makes potential therapeutic options easier [12], their reproducibility in individual examiners has been challenged and remains to be discussed, and a more precise anatomic distinction between them should be more clarified with further efforts.

Colposcopy has been considered a visual procedure that is dependent on observer experience. However, this study shows that when the 2011 IFCPC classification is used, colposcopic accuracy by examiners with differing amounts of experience has no significant difference. Similar conclusions were obtained for the 2002 classification in an online quality assurance program for colposcopy in Italy [15] and Hammes et al.'s study [14]. Therefore, regardless of experience, the reproducibility of the colposcopic impression, when performed by trained colposcopists with high-quality classification, is higher than is generally thought [15].

The new colposcopic nomenclature provides standardized interpretations of the colposcopic findings and represents the latest knowledge in this area. Although the reproducibility of TZ and a few signs of predictive value remain to be discussed, the present study confirms that colposcopy according to the 2011 IFCPC classification in trained colposcopists is a potential screening method. However, there was some bias in our study. Because this is a retrospective study, the Reid index and Swede Score were defined from colpophotographs and are not representative of real life colposcopy. There is also potential bias by colposcopists and pathologists due to unblinded cytologic and HPV test results. And in a critical sense, it is not a colposcopic index; how to make it measurable and quantitative should be investigated in the future [17].

## Figures and Tables

**Table 1:** Agreement between colposcopic diagnosis and cervical histopathology.

Colposcopic diagnosis	Histopathological diagnosis (N)				
	Normal or benign	LSIL	HSIL	Carcinoma	Total
IFCPC classification					
Normal or benign	190^*∗*^	41^*∗∗*^	9	0	240
LSIL	87^*∗∗*^	91^*∗*^	27^*∗∗*^	0	205
HSIL	6	11^*∗∗*^	56^*∗*^	0^*∗∗*^	73
Carcinoma	0	0	3^*∗∗*^	4^*∗*^	7
*Total*	*283*	*143*	*95*	*4*	*525*

RCI					
Normal or benign	202^*∗*^	69^*∗∗*^	22	0	293
LSIL	72^*∗∗*^	64^*∗*^	39^*∗∗*^	0	175
HSIL	9	10^*∗∗*^	34^*∗*^	4^*∗∗*^	57
Carcinoma	0	0	0^*∗∗*^	0^*∗*^	0
*Total*	*283*	*143*	*95*	*4*	*525*

Modified RCI					
Normal or benign	8^*∗*^	0^*∗∗*^	1	0	9
LSIL	248^*∗∗*^	115^*∗*^	44^*∗∗*^	0	407
HSIL	27	28^*∗∗*^	50^*∗*^	4^*∗∗*^	109
Carcinoma	0	0	0^*∗∗*^	0^*∗*^	0
*Total*	*283*	*143*	*95*	*4*	*525*

Swede Score					
Normal or benign	260^*∗*^	116^*∗∗*^	49	0	425
LSIL	20^*∗∗*^	24^*∗*^	36^*∗∗*^	1	81
HSIL	3	3^*∗∗*^	10^*∗*^	3^*∗∗*^	19
Carcinoma	0	0	0^*∗∗*^	0^*∗*^	0
*Total*	*283*	*143*	*95*	*4*	*525*

^*∗*^Perfect agreement; ^*∗∗*^agreement within one grade.

**Table 2:** Correlation of colposcopic findings in 2011 IFCPC nomenclature with histological diagnoses and their PPVs.

Colposcopic finding	Histological diagnoses (N)				Total (%)	PPV (%)	
	Normal or benign	LSIL	HSIL	Carcinoma		LSIL+ (95% CI)	HSIL+ (95% CI)
Minor changes							
Thin AWE^*∗*^	121	89	53	0	263	54.0%	20.2%
	(46.0)	(33.8)	(20.2)	(0.0)	(100.0)	(47.9%–60.1%)	(15.3%–25.0%)
Fine mosaic	3	12	10	0	25	88.0%	40.0%
	(12.0)	(48.0)	(40.0)	(0.0)	(100.0)	(74.3%–100%)	(19.4%–60.6%)
Fine punctuation	10	11	10	0	31	67.7%	32.3%
	(32.3%)	(35.5)	(32.3)	(0.0)	(100.0)	(50.3%–85.2%)	(14.8%–49.7%)

Major changes							
Dense AWE^*∗*^	9	8	43	3	63	85.7%	73.0%
	(14.3)	(12.7)	(68.3)	(4.8)	(100.0)	(76.8%–94.6%)	(61.7%–84.3%)
Coarse mosaic	3	3	18	2	26	88.5%	76.9%
	(11.5)	(11.5)	(69.2)	(7.7)	(100.0)	(75.3%–100%)	(59.6%–94.3%)
Coarse punctuation	3	3	10	1	17	82.4%	64.7%
	(17.6)	(17.6)	(58.8)	(5.9)	(100.0)	(62.1%–100%)	(39.4%–90.0%)
Sharp border	3	3	5	2	13	76.9%	53.8%
	(23.1)	(23.1)	(38.5)	(15.4)	(100.0)	(50.4%–100%)	(22.5%–85.2%)
Inner border sign	0	0	4	0	4	100.0%	100.0%
	(0.0)	(0.0)	(100.0)	(0.0)	(100.0)		
Ridge sign	0	0	5	2	7	100.0%	100.0%
	(0.0)	(0.0)	(71.4)	(28.6)	(100.0)		

Nonspecific							
Iodine negativity	167	104	71	2	344	51.5%	21.2%
	(48.5)	(30.2)	(20.6)	(5.8)	(100.0)	(46.1%–56.8%)	(16.9%–25.6%)

Suspicious for invasion							
Atypical vessels	0	0	4	4	8	100.0%	100.0%
	(0.0)	(0.0)	(50.0)	(50.0)	(100.0)		
Additional signs	0	0	1	2	3	100.0%	100.0%
	(0.0)	(0.0)	(33.3)	(66.7)	(100.0)		

Miscellaneous							
Condyloma	0	11	0	0	11	100.0%	0.0%
	(0.0)	(100.0)	(0.0)	(0.0)	(100.0)		
Polyp	20	6	5	0	31	35.5%	16.1%
	(64.5)	(19.4)	(16.1)	(0.0)	(100.0)	(17.6%–53.3%)	(2.4%–29.8%)
Contact bleeding	5	1	1	1	8	37.5%	25.0%
	(62.5)	(12.5)	(12.5)	(12.5)	(100.0)	(0.0%–80.8%)	(0.0%–63.7%)

Total	344	251	240	19	854	—	—
	(40.3)	(29.4)	(28.1)	(2.2)	(100.0)		

^*∗*^AWE: acetowhite epithelium.

**Table 3:** Agreements between colposcopy and histopathology in examiners with different experience according to the IFCPC classification.

Working experience of colposcopists	Colposcopic diagnosis and pathological diagnosis (N)			
	Matched	Unmatched	Total	Agreement (%)
More than 10 years	119	64	183	65.0
5~10 years	124	78	202	61.4
Less than 5 years	98	42	140	70.0

Total	341	184	525	64.95

**Table 4:** Frequency of transformation zone types in nomenclature among 13 colposcopists.

Colposcopist	Transformation zone			Total (%)
	Type 1	Type 2	Type 3	
Colposcopist A	5 (8.47)	0 (0.00)	54 (91.53)	59 (100.0)
Colposcopist B	1 (6.25)	3 (18.75)	12 (75.00)	16 (100.0)
Colposcopist C	2 (8.00)	1 (4.00)	22 (88.00)	25 (100.0)
Colposcopist D	7 (35.00)	2 (10.00)	11 (55.00)	20 (100.0)
Colposcopist E	4 (12.50)	3 (9.38)	25 (78.13)	32 (100.0)
Colposcopist F	11 (34.38)	2 (6.25)	19 (59.38)	32 (100.0)
Colposcopist G	2 (10.00)	4 (20.00)	14 (70.00)	20 (100.0)
Colposcopist H	16 (27.59)	7 (12.07)	35 (60.34)	58 (100.0)
Colposcopist I	11 (23.40)	4 (8.51)	32 (68.09)	47 (100.0)
Colposcopist J	39 (29.55)	3 (2.27)	90 (68.18)	132 (100.0)
Colposcopist K	8 (24.24)	3 (9.09)	22 (66.67)	33 (100.0)
Colposcopist L	7 (20.00)	4 (11.43)	24 (68.57)	35 (100.0)
Colposcopist M	4 (25.00)	2 (12.50)	10 (62.50)	16 (100.0)

Total	117 (22.29)	38 (7.24)	370 (70.48)	525 (100.0)

**Table 5:** Accuracy of colposcopic diagnosis in distinguishing cervical histopathology at different cutoffs.

	Compared with cervical histopathology (gold standard)						
	Validity		Predictability		Comprehensiveness		
	Sensitivity	Specificity	PPV	NPV	YI	PLR	NLR
IFCPC terminology							
HSIL+	63.64%	96.01%	78.75%	91.91%	0.596	15.95	0.38
	*(54.0%–73.3%)*	*(94.1%–97.9%)*	*(69.6%–87.9%)*	*(89.4%–94.5%)*			
LSIL+	79.34%	67.14%	67.37%	79.17%	0.465	2.41	0.31
	*(74.2%–84.5%)*	*(61.6%–72.6%)*	*(61.9%–72.8%)*	*(74.0%–84.3%)*			

RCI							
HSIL+	38.38%	95.54%	66.67%	86.97%	0.339	8.61	0.65
	*(28.6%–48.1%)*	*(93.6%–97.5%)*	*(54.0%–79.3%)*	*(83.9%–90.0%)*			
LSIL+	62.40%	71.38%	65.09%	68.94%	0.338	2.18	0.53
	*(56.3%–68.5%)*	*(66.1%–76.7%)*	*(58.9%–71.3%)*	*(63.6%–74.3%)*			

Modified RCI							
HSIL+	54.55%	87.09%	49.54%	89.18%	0.416	4.22	0.52
	*(44.6%–64.5%)*	*(83.9%–90.3%)*	*(40.0%–59.1%)*	*(86.2%–92.2%)*			
LSIL+	99.6%	2.83%	46.71%	88.89%	0.024	1.02	0.15
	*(98.8%–100.0%)*	*(0.9%–4.8%)*	*(42.4%–51.0%)*	*(63.3%–100.0%)*			

Swede Score							
HSIL+	13.13%	98.59%	68.42%	83%	0.117	9.32	0.88
	*(6.4%–19.9%)*	*(97.5%–99.7%)*	*(45.4%–91.4%)*	*(79.7%–86.3%)*			
LSIL+	31.82%	91.87%	77%	61.18%	0.237	3.92	0.74
	*(25.9%–37.7%)*	*(88.7%–95.1%)*	*(68.6%–85.4%)*	*(56.5%–65.8%)*			

**Table 6:** Comparison between IFCPC classification and the RCI, modified RCI, and Swede Score.

	IFCPC classification (N)		
	LSIL−	HSIL+	Total
RCI			
LSIL−	8	31	39
HSIL+	437	49	486
*Total*	*445*	*80*	*525*

Modified RCI			
LSIL−	398	18	416
HSIL+	47	62	109
*Total*	*445*	*80*	*525*

Swede Score			
LSIL−	444	62	506
HSIL+	1	18	19
*Total*	*445*	*80*	*525*
